# Transcriptional repression of cancer stem cell marker CD133 by tumor suppressor p53

**DOI:** 10.1038/cddis.2015.313

**Published:** 2015-11-05

**Authors:** E K Park, J C Lee, J W Park, S Y Bang, S A Yi, B K Kim, J H Park, S H Kwon, J S You, S W Nam, E J Cho, J W Han

**Affiliations:** 1 Department of Biochemistry and Molecular Biology, Research Center for Epigenome Regulation, School of Pharmacy, Sungkyunkwan University, Suwon, Republic of Korea; 2 Division of Cardiology, Department of Medicine, Stanford University School of Medicine, 265 Campus Drive, Room G1120B, Stanford, CA, USA; 3 College of Pharmacy, Yonsei Institute of Pharmaceutical Sciences, Yonsei University, Incheon, Republic of Korea; 4 Department of Biochemistry, School of Medicine, Konkuk University, Seoul, Republic of Korea; 5 Department of Pathology, College of Medicine, The Catholic University of Korea, Seoul, Republic of Korea

## Abstract

Novel therapeutic strategies are needed to overcome cancer recurrence, metastasis, and resistance to chemo- and radiotherapy. Cancer stem cells (CSCs) are major contributors to the malignant transformation of cells due to their capacity for self-renewal. Although various CSC markers have been identified in several types of tumors, they are primarily used as cancer-prediction markers and for the isolation of CSC populations. CD133, one of the best-characterized CSC markers in distinct solid tumor types, was shown to be correlated with CSC tumor-initiating capacity; however, the regulation of CD133 expression and its function in cancer are poorly understood. Here, we show that CD133 expression is negatively regulated by direct binding of the p53 tumor suppressor protein to a noncanonical p53-binding sequence in the CD133 promoter. Binding of p53 recruits Histone Deacetylase 1 (HDAC1) to the CD133 promoter and subsequently suppresses CD133 expression by reducing histone H3 acetylation. Furthermore, CD133 depletion suppresses tumor cell proliferation, colony formation, and the expression of core stemness transcription factors including NANOG, octamer-binding transcription factor 4 (OCT4), SOX2, and c-MYC. Critically, the anti-proliferative effects of p53 are antagonized by rescue of CD133 expression in a p53 overexpressing cell line, indicating that the tumor suppressive activity of p53 might be mediated by CD133 suppression. Taken together, our results suggest that p53-mediated transcriptional regulation of CD133 is a key underlying mechanism for controlling the growth and tumor-initiating capacity of CSCs and provide a novel perspective on targeting CSCs for cancer therapy.

Cancer stem cells (CSCs) are a rare population of tumor cells that maintain tumor initiation and self-renewal capacity. According to the cancer stem cell model, a subpopulation of cells with stem cell-like characteristics exists in tumors, and these cells give rise to the bulk of the tumor.^1, 2^ Given the rapid advances in CSC research, there is a renewed optimism for the development of innovative cancer therapies that can target CSCs in bulk tumors.^3, 4^ Several recent studies have identified CSC markers that are uniquely expressed in CSCs and thus provide possible targets for CSC therapy.^4, 5^

A number of cell surface markers, CD133 (Human prominin-1, PROM1), CD44, and CD24, have been used to isolate CSC populations.^6, 7, 8, 9^ CD133 is a pentaspan transmembrane cell-surface protein that is primarily localized to the plasma membrane.^6, 10^ Although it was originally identified as a marker for CD34^+^ hematopoietic progenitor cells^11^ and neuroepithelial stem cells,^12^ CD133 is also expressed in cancer progenitor cells in the brain, breast, prostate, liver, ovary, lung, colon, and pancreas.^7, 13, 14, 15, 16, 17, 18, 19^ Furthermore, recent studies have shown that CD133 can serve as an essential marker for detecting and enriching several types of CSCs and that CD133 expression levels are correlated with CSC tumor-promoting capacity.^15, 20^ Although these results strongly suggested that CD133 could be a potential marker for CSCs, its precise functions and regulation in CSCs remained unclear.

The potent tumor suppressor protein 53 (p53) is involved in several cellular processes including metabolic homeostasis, deoxyribonucleic acid (DNA) repair, growth arrest, senescence, and apoptosis.^21, 22, 23^ Specifically, p53 lies at the center of the stress response pathways that prevent tumor cell growth and survival.^24^ As a key transcription factor, p53 regulates the transcription of several target genes that are related to various pathways.^24, 25^ Recently, a study demonstrated that p53 could directly suppress CD44 expression, and this suppression inhibited the growth and tumor-initiating capacity of highly tumorigenic mammary epithelial cells.^26^ Moreover, p53 could repress the expression of various target genes such as NANOG that contribute to the maintenance of the tumor-initiating cell pool.^25, 27^ These results support the idea that p53 could be a possible regulator of CSC tumorigenic function by modulating the expression of CSC-specific genes.

Here we identify CD133 as a novel p53 target gene. By directly binding to the CD133 promoter, p53 suppresses the CD133 transcription. A putative p53-binding sequence within the CD133 promoter is essential for p53-binding and the subsequent recruitment of HDAC1 to facilitate epigenetic modifications of the CD133 promoter. Ultimately, this cascade leads to the inhibition of CD133 transcription. This p53-based restriction of CD133 expression can be observed in several cancer cell lines under normal and stress conditions, and rescue of CD133 expression abolishes the tumor suppressive effect of p53 in highly tumorigenic cancer lines. Collectively, p53-mediated CD133 inhibition is a critical regulatory mechanism for preventing cancer survival and tumorigenesis.

## Results

### Inverse correlation of p53 tumor suppressor and CD133 expression in cancer

**Figure 1:**
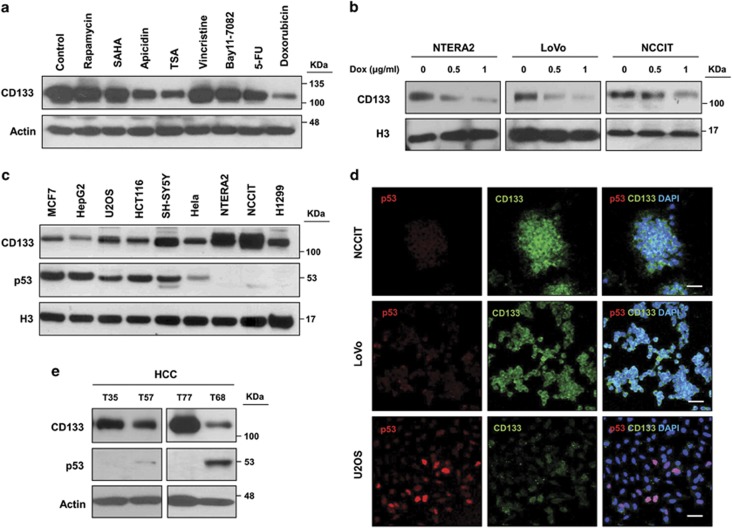
Inverse correlation of p53 tumor suppressor and CD133 expression in cancers. (**a**) Western blot of CD133 expression in NTERA2 cells exposed to cytotoxic drugs (1 *μ*M of rapamycin, apicidin, vincristine, and Bay 11–7082, 10 nM of SAHA, 1 *μ*g/ml TSA, 5-fluorouracil (5-FU) and Dox) for 24 h. (**b**) CD133 protein expression levels in NTERA2, LoVo, and NCCIT cells exposed to the indicated dose of Dox. (**c**) CD133 and p53 protein expression levels were determined by immunoblotting for nine cancer cell lines. (**d**) Immunofluorescence detection of CD133, p53, and DAPI in NCCIT, LoVo, and U2OS cell lines. Scale bar, 50 *μ*m. (**e**) CD133 and p53 expression levels in hepatocellular carcinoma were determined in four patient samples by immunoblotting

Cell-based phenotypic assays and pathway screening of synthetic small molecules and natural products have historically provided useful chemical tools for modulating and/or studying complex cellular processes. To unravel the mechanism regulating CD133 expression, we first screened small molecules including mTOR inhibitor (rapamycin), I*κ*B*α* inhibitor (Bay 11-7082), Histone deacetylase (HDAC) inhibitors (apicidin, trichostatin A (TSA), and suberoylanilide hydroxamic acid (SAHA)), clinical anti-cancer agents (Doxorubicin (Dox) and 5-fluorouracil (5-FU)), and an inhibitor of microtubule formation (vincristine) on CD133 expression in human embryonic carcinoma NTERA2 cells. Among the tested molecules, Dox showed the most significant downregulation of CD133 expression (Figure 1a). To further characterize the effects of Dox on CD133 expression, three different cancer cell lines, NCCIT, human embryonic carcinoma NTERA2, and colon adenocarcinoma LoVo, were treated with varying doses of Dox. In these cell lines, Dox treatment markedly suppressed CD133 expression in a dose-dependent manner (Figure 1b). To test the possible involvement of p53 in Dox-mediated suppression of CD133 expression, we examined the expression patterns of p53 and CD133 in 28 established cancer cell lines carrying wild-type (WT) p53 or various p53 mutants (Supplementary Table 1). Immunoblot analysis showed that CD133 was highly expressed in NTERA2 and NCCIT cells (Figure 1c and Supplementary Figure 1), which are derived from poorly differentiated germ cell tumors and utilized as CSC model cell lines.^28^ Although the p53 expression levels differed greatly among the various cancer cell lines, p53 expression was inversely correlated with CD133 expression. In particular, cell lines such as NTERA2, NCCIT, and LoVo, where CD133 was highly expressed, showed significantly reduced p53 expression (Figure 1c and Supplementary Figure 1). In contrast, U2OS, Michigan Cancer Foundation-7 (MCF7), and HepG2 cells, which express low levels of CD133, had high p53 protein levels. Consistent with this result, CD133 and p53 immunofluorescence staining (IF) confirmed their inverse expression pattern in NCCIT, LoVo, and U2OS cells (Figure 1d). Importantly, immunoblot analysis of hepatocellular carcinoma (HCC) tissue samples obtained from four different patients also revealed that patients exhibiting high p53 protein levels had relatively low CD133 protein levels (Figure 1e), demonstrating the inverse expression pattern of p53 and CD133 *in vivo* human cancer tissues. These results indicate that p53 could regulate CD133 expression or *vice versa*.

### CD133 downregulation by stress-induced activation of p53

**Figure 2:**
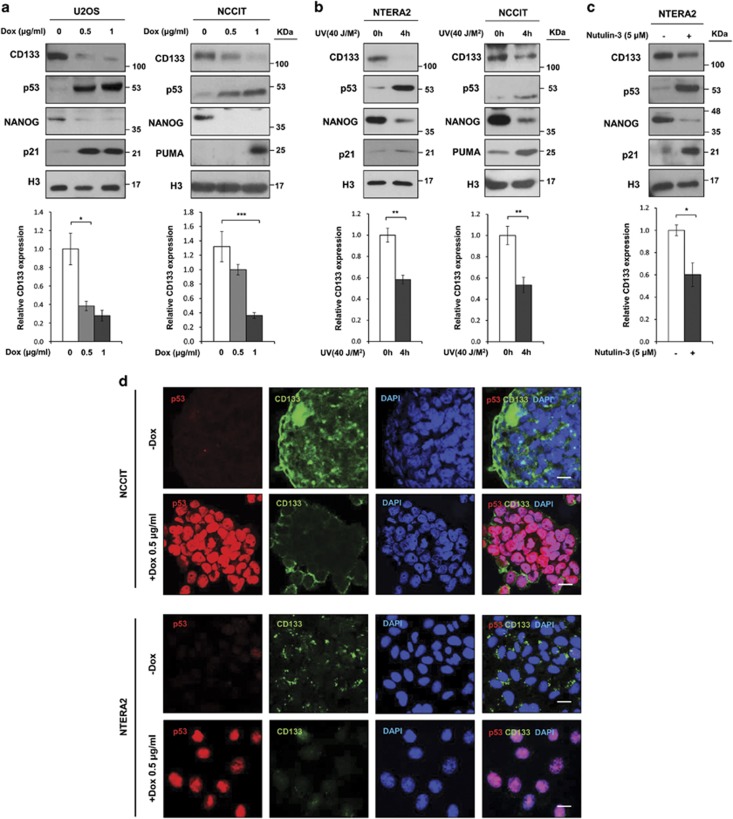
Downregulation of CD133 by p53 activation. (**a**) mRNA levels and protein expression levels in cell lysates from U2OS and NCCIT treated with the indicated dose of Dox. (**b**) Induction of p53 protein after UV irradiation (40 J/M^2^) downregulates CD133 mRNA and protein levels in NTERA2 and NCCIT cells. (**c**) Treatment with Nutlin-3 (5 *μ*M) downregulated CD133 mRNA and protein expression in NTERA2 cells. (**d**) Immunofluorescence detection of CD133, p53, and DAPI in NCCIT and NTERA2 cells after Dox treatment (0.5 *μ*g/ml, 24 h). Scale bar, 25 *μ*m. The error bars represent the mean±S.D. *n*=3. *P<0.05, **P<0.01, and ***P<0.001

To examine the influence of p53 on CD133 expression, we tested whether the induction of p53 affected CD133 expression. Following treatment with Dox, a potent genotoxic agent, p53 expression levels increased and CD133 levels decreased in a dose-dependent manner (Figure 2a). Consistent with this data, increased p53 expression due to ultraviolet (UV) irradiation or treatment with Nutlin-3, a specific inhibitor of MDM2-binding to p53, known to induce p53 stabilization, downregulated CD133 expression in NTERA2 and NCCIT cells (Figures 2b and c). As expected, the induction of p53 in response to Dox, UV, or Nutullin-3 led to the expression of p53 positive target genes such as p21 or PUMA, and the suppression of p53 repressive target gene, NANOG (Figures 2a and c). IF assays also demonstrated the impaired expression of CD133 in response to Dox treatment in NCCIT and NTERA2 cells (Figure 2d). To further determine whether p53 affects CD133 expression at the transcriptional or posttranscriptional levels, we measured CD133 messenger RNA (mRNA) levels using quantitative reverse transcription polymerase chain reaction (qRT-PCR), finding that CD133 mRNA levels were also decreased by Dox, UV, and Nutlin-3 treatments (Figures 2a and c). This result indicates that p53 pathway activation is related to the suppression of CD133 transcription.

### Requirement of p53 for suppressing CD133 expression

**Figure 3:**
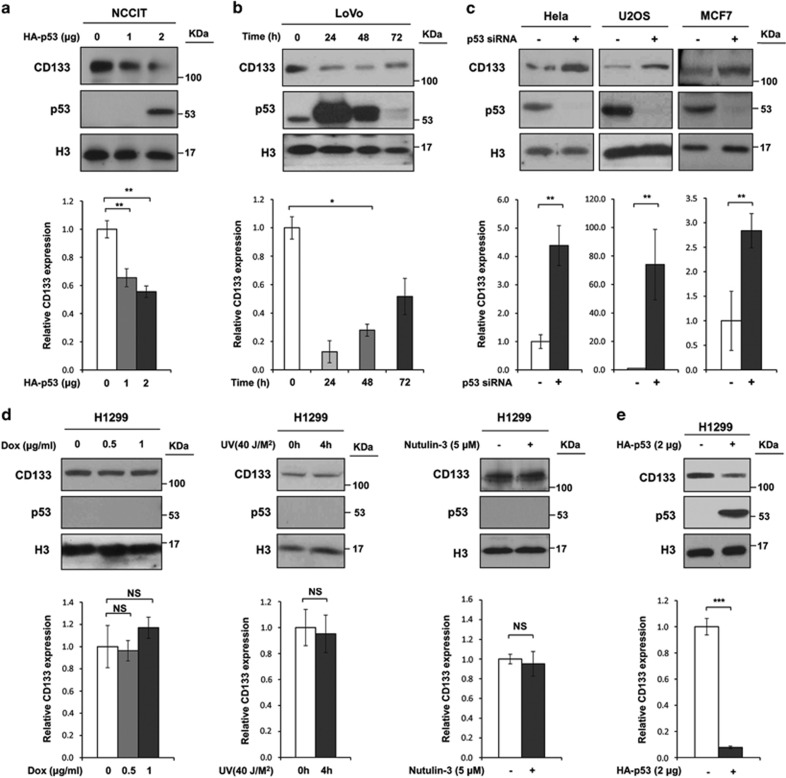
Requirement of p53 for suppressing CD133 expression. (**a**) Western blot and qPCR analyses were performed using lysates of NCCIT cells that had been transfected with the indicated amounts of HA-p53-expressing or empty vector. (**b**) Western blot and qPCR analyses were performed using lysates of LoVo cells transfected with HA-p53-expressing vector for the indicated time. (**c**) Western blot and qPCR analyses were performed for various cancer cell lines that had been transfected with either CD133 siRNA or scramble siRNA-expressing vector. (**d**) mRNA and protein expression levels of H1299 cells treated with the indicated dose of Dox (left panel), after UV irradiation (40 J/M^2^; middle panel), or treatment with Nutlin-3 (5 *μ*M; right panel). (**e**) Western blot and qPCR analyses were performed using lysates of H1299 cells that had been transfected with the indicated amounts of HA-p53-expressing or empty vector. The error bars represent the mean±S.D. *n*=3. *P<0.05, **P<0.01, and ***P<0.001; NS, not significant

We next tested whether p53 is essential for suppressing CD133 expression under normal growth conditions. Ectopic expression of WT p53 but not mutant p53 (p.R175H) with impaired DNA-binding affinity^29, 30^ decreased CD133 protein and mRNA levels in NCCIT and LoVo cells, which had high CD133 expression under normal culture conditions (Figures 3a and b and Supplementary Figure 2). Moreover, the reduction in CD133 was rescued to the baseline level as ectopic p53 expression in LoVo cells was cleared over time in culture (Figure 3b). Furthermore, using short interfering RNA (siRNA) targeting p53, we found that depletion of p53 in several cancer cell lines in which p53 protein levels were relatively high (HeLa, U2OS, and MCF7), led to an increase in CD133 protein and mRNA levels (Figure 3c). In contrast, CD133 expression was not affected by Dox, Nutlin-3 treatment or UV irradiation in H1299 cells possessing a homozygous partial deletion of the p53 gene that consequently do not express the p53 tumor suppressor protein (Figure 3d), whereas ectopic p53 expression reduced CD133 protein and mRNA levels (Figure 3e). These results indicate that p53 is essential for the downregulation of CD133 expression.

### Transcriptional regulation of CD133 by p53

**Figure 4:**
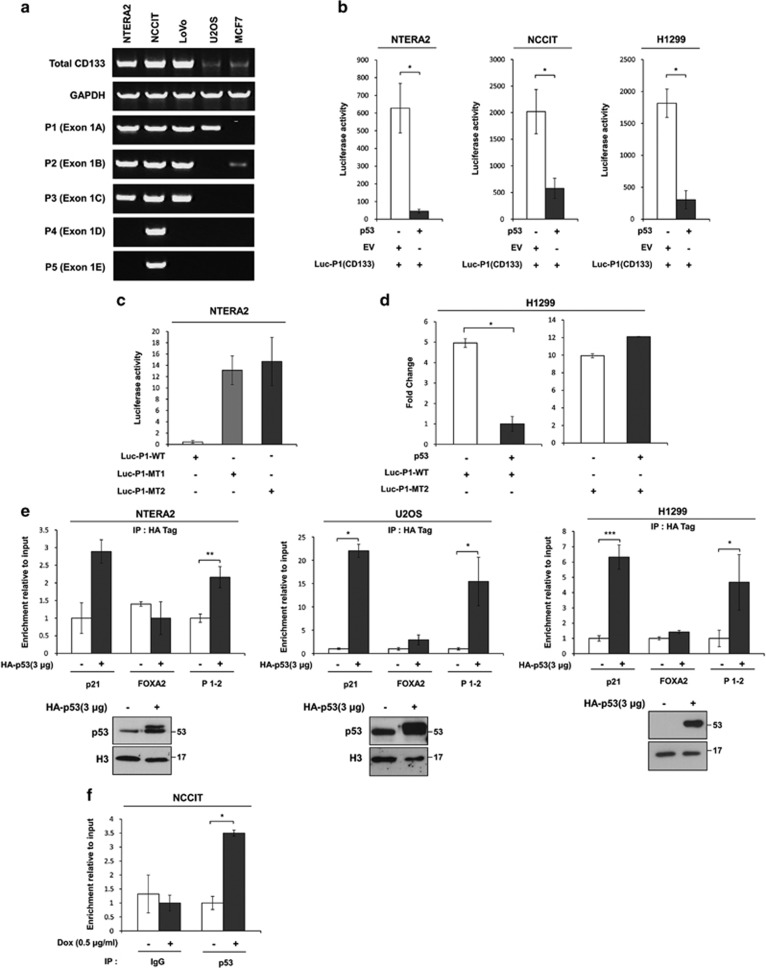
p53 directly regulates CD133 transcription. (**a**) Transcript variants produced by the alternate activity of five promoters (promoter 1–5) were identified in five cell lines by nested PCR. (**b**) p53-mediated repression of CD133 promoter activity in NTERA2, NCCIT, and H1299 cells. (**c**) Activity of the control CD133 promoter and two mutated CD133 promoters in NTERA2 cells. (**d**) Activity of the control CD133 promoter and mutated CD133 promoter in H1299 cell lines. Empty or p53 expression vectors were co-transfected with the reporter gene vector. (**e**) ChIP analysis of p53-binding to CD133 promoter DNA in NTERA2, U2OS, and H1299 cells that had been transfected with either control or p53 expression vector. Protein levels of ectopically expressed p53 were examined in total ChIP lysates. (**f**) ChIP analysis of p53-binding to CD133 promoter DNA in NCCIT cells treated with Dox or DMSO. The error bars represent the mean±S.D. *n*=3. *P<0.05, **P<0.01 and ***P<0.001; NS, not significant

CD133 has five different promoters in the 5′ untranslated region (promoter 1 (P1), promoter 2 (P2), promoter 2 (P3), promoter 4 (P4), and promoter 5 (P5)),^31^ resulting in alternatively spliced CD133 variants (Supplementary Figure 3a). To determine which promoter is active in each cancer cell line, we examined the expression of each CD133 variants using nested polymerase chain reaction (PCR) amplification with a specific primer for each genomic isoform. NCCIT cells utilized all five promoters while NTERA2 and LoVo cells employed three promoters, P1, P2, and P3. U2OS and MCF7 utilized only one promoter each, P1 and P2, respectively. Consistent with the promoter activity in each cell lines, CD133 was highly expressed in NTERA2, NCCIT, and LoVo cells but relatively less expressed in U2OS and MCF7 cells (Figure 4a).

As the majority of cancer cell lines (except MCF7) utilized promoter P1, we examined the effects of p53 on promoter P1 using a luciferase construct containing the full-length promoter for Exon 1A (Luc-P1). Consistent with the downregulation of CD133 expression by ectopic p53 expression (Figures 3a, b and e), Luc-P1 luciferase activity was severely impaired by ectopic p53 expression in NTERA2, NCCIT, and H1299 cells, indicating that P1 is a p53 target (Figure 4b).

Next, we investigated whether p53-mediated CD133 inhibition resulted from direct binding of p53 to the CD133 promoter. Sequence analysis determined that the −700 bp region of promoter P1 contained a unique sequence with strong similarity to a noncanonical p53-binding sequence, which consisted of four p53-binding sites in an alternating head-to-tail arrangement (Supplementary Figure 3b).^32^ Interestingly, the unique sequence found in promoter P1 is analogous to the p53-binding sequence observed in the promoter of another CSC marker, CD44 (Supplementary Figure 3b).^26^ Using site-directed mutagenesis, we generated a mutant type (MT) P1 promoter in which cytosine was replaced with either adenine (C→A) or thymine (C→T) (Supplementary Figure 3b). Luciferase assays showed that the reporter activity of Luc-P1-MT was higher than that of Luc-P1-WT (Figure 4c). Moreover, the reporter activity of Luc-P1-WT was reduced by ectopic p53 expression or Dox treatment while Luc-P1-MT luciferase activity was unaffected (Figure 4d and Supplementary Figure 3c). These data indicate that the putative p53-binding sequence is essential for p53-mediated suppression of CD133.

To determine whether p53 directly binds to the P1 promoter region containing the noncanonical p53-binding sequence, we analyzed p53 enrichment at promoter P1. Chromatin immunoprecipitation coupled to qPCR (ChIP-qPCR) assays showed that ectopically expressed p53 bound directly to the P1 promoter in NTERA2, U2OS, and H1299 cells (Figure 4e). The p53 enrichment on the CD133 promoter was comparable to that on the positive control promoter (p21 promoter), but not to that on the negative control promoter (FOXA2 promoter) (Figure 4e). Furthermore, dox-induced endogenous p53 was also recruited to the P1 promoter, indicating that direct binding of p53 to the CD133 promoter is required for p53-mediated suppression of CD133 (Figure 4f).

As previous studies showed that recruitment of HDAC1 to the target promoter is necessary for p53-mediated gene silencing,^33, 34^ we tested whether HDAC1 is recruited to the CD133 promoter with p53. ChIP-qPCR with HDAC1 revealed that ectopic p53 expression increased HDAC1 recruitment to the P1 promoter, which then in turn decreased H3 acetylation (Supplementary Figure 4a). These results were consistent with the co-immunoprecipitation of endogenous p53 and HDAC1 in Hela cell line (Supplementary Figure 4b). Furthermore, ectopic p53 expression also increased the levels of known repressive histone marker H3K9me3 (Supplementary Figure 4a). Taken together, these data indicate that p53 negatively regulates CD133 transcription through direct binding to the CD133 P1 promoter.

### CD133 is required for tumor growth and survival

**Figure 5:**
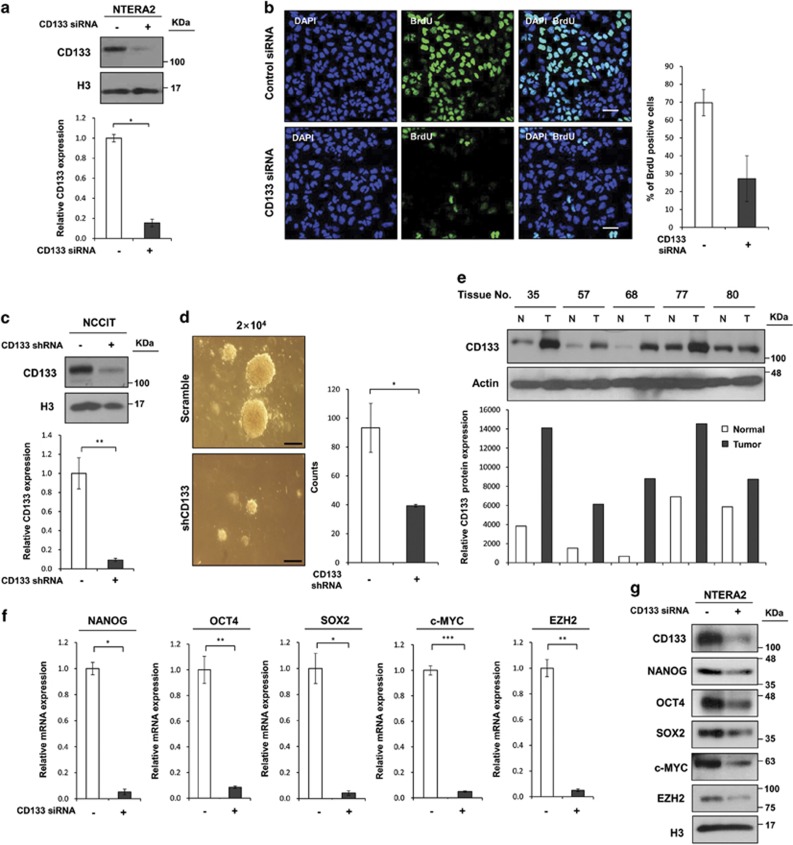
CD133 is required for stem cell-like characteristics and tumor growth. (**a**) Immunoblot and qRT-PCR assays for CD133 expression in NTERA2 cells that had been transiently transfected with CD133-specific siRNA. (**b**) BrdU assays were performed to assess CD133 proliferation capacity in NTERA2 control and CD133 knockdown cells. Scale bar, 50 *μ*m. (**c**) Immunoblot and qRT-PCR analysis of CD133 expression levels in NCCIT cells that had been infected with lentiviruses expressing CD133-specific shRNA. (**d**) Soft agar colony formation by control and CD133 knockdown stable NCCIT cells. Two weeks after seeding, the number of colonies was counted (2 × 10^4^ cells). Scale bar, 250 *μ*m. (**e**) Immunoblot analysis of protein samples from five patients human hepatocellular carcinoma and normal tissues to measure CD133 protein levels. (**f**) qRT-PCR for NANOG, OCT4, SOX2, c-MYC, and EZH2 in NTERA2 cells transfected with CD133-specific siRNA. (**g**) Expression levels of CD133, NANOG, OCT4, SOX2, c-MYC, and EZH2 proteins in NTERA2 cells that had been transfected with CD133-specific siRNA. The error bars represent the mean±S.D. *n*=3. *P<0.05, **P<0.01, and ***P<0.001

Although CD133 is a putative marker for CSCs in many distinct solid tumor types and is associated with aggressive cancers and poor prognosis, the precise function of CD133 in tumor formation remained unknown. We first examined whether CD133 expression is essential for NTERA2 tumor cell growth by measuring bromodeoxyuridine (BrdU) incorporation in cells transfected with CD133 siRNA (Figure 5a). Twenty-six percent of the CD133 knockdown cells were BrdU positive while 70% of the control cells were BrdU positive (Figure 5b); these data were corroborated in NCCIT cells (Supplementary Figure 5a).

To further address the role of CD133 in anchorage-independent growth, we analyzed the soft agar colony formation efficiency of a stable NCCIT cell line that continuously expressed short hairpin RNA (shRNA) targeting CD133. Endogenous CD133 expression was successfully abrogated in the CD133 stable cell line (Figure 5c), and CD133 depletion led to a reduced efficiency of soft agar colony formation, with 93±4 colonies for the control line *versus* 39±0.5 colonies for the knockdown line (Figure 5d and Supplementary Figure 5b), indicating that CD133 might have a critical role in the malignant transformation of cells. Consistent with this result, CD133 levels in tumor tissue are greater than those in normal tissue from the same liver carcinoma patients (Figure 5e).

CD133 expression is accompanied by the expression of core stemness transcription factors such as NANOG, octamer-binding transcription factor 4 (OCT4), SOX2, and c-MYC, which have been proposed to have important roles in cancer reoccurrence, tumor growth, and metastasis.^35, 36, 37, 38^ Another stemness marker, Enhancer of zeste homolog 2 (EZH2), has also been shown to promote the neoplastic transformation of breast epithelial cells.^39, 40^ To test whether CD133 is involved in the expression of these genes, their mRNA and protein levels were measured in cell lines that had been transfected with CD133 siRNA. Interestingly, CD133 depletion suppressed the expression of all of the core stemness genes and EZH2 in NTERA2 cells (Figures 5f and g), while the expression of other cancer-related genes such as hTERT^41, 42^ and DAB21P^43^ was not affected (Supplementary Figure 5c). Collectively, these results indicate that CD133 might have an essential role in the transcription of stemness genes, and suggest that CD133 expression is responsible for CSC growth and survival through the maintenance of stem cell-like features.

### CD133 inhibition is required for p53 tumor-suppressive activity

**Figure 6:**
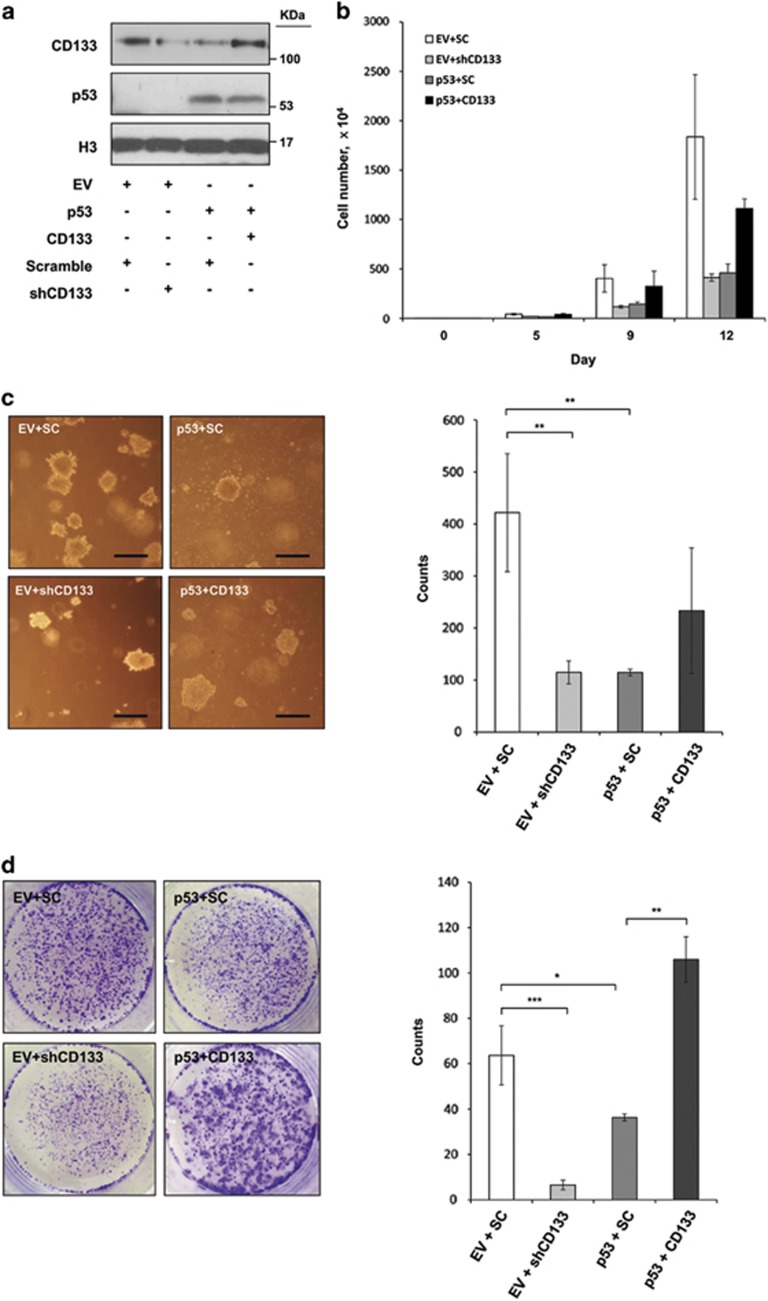
p53-based effects on tumor growth depend on suppression of CD133. (**a**) NCCIT cells were stably transfected with lentivirus for stable expression of CD133 shRNA (lane 2), retrovirus for stable expression of p53 (lane 3) or co-transfected with p53 and CD133 stable expression vector for rescue (lane 4). Expression levels were analyzed by immunoblotting after the establishment of stable cell lines. (**b**) Growth of NCCIT cells that had been stably infected with lentiviral or retroviral constructs expressing CD133 shRNA, or stably expressing p53 and the CD133 vector. (**c**) Soft agar colony formation by NCCIT cells that had been stably infected with lentiviral or retroviral constructs expressing CD133 shRNA or the p53 and CD133 vector; the number of colonies was counted. (**d**) Adherent colony formation assays were performed by seeding NCCIT cells that had been stably infected with lentiviral or retroviral constructs expressing shRNA or stably expressing p53 and CD133 vector. Scale bar, 250 *μ*m. The error bars represent the mean±S.D. *n*=3. *P<0.05, **P<0.01, and ***P<0.001

To investigate whether p53-mediated inhibition of CD133 is necessary for p53 tumor suppressive activity, we utilized an ectopic CD133 expression construct containing only the CD133 mRNA coding DNA sequence (CDS). Ectopic p53 expression reduced endogenous CD133 levels (Figure 6a, lane 3), but no differences were observed in the expression of exogenous CD133 (Figure 6a, lane 4), indicating that the CD133 promoter region is required for p53-mediated inhibition of CD133 transcription. Compared with control cells, ectopic p53 expression reduced the growth rate of NCCIT cells to that observed for the CD133 knockdown stable cell line (Figure 6b); however, the effects of exogenous p53 on growth rate suppression were diminished by CD133 overexpression. These data indicate that abrogation of CD133 is required for p53-mediated cell cycle arrest. In accordance with this data, ectopic p53 expression or CD133 shRNA resulted in a reduced efficiency of soft agar colony formation, while no significant decrease was observed in the number of colonies formed by co-overexpression of CD133 with ectopic p53 (Figure 6c). Moreover, the results of the adherent colony formation assay were similar, indicating that CD133 suppression is required for p53 tumor suppressive activity under normal culture conditions (Figure 6d).

## Discussion

CSCs have been proposed as the driving force of tumorigenesis and metastasis as the term CSC refers to a subset of tumor cells that possess potential for self-renewal and multilineage differentiation. Over the past decade, several CSC markers have been identified in a wide range of solid and hematopoietic tumors.^4, 44^ CD133 is the most well-known marker used for CSC identification and isolation. Many studies have shown that CD133^+^ cells possess stemness properties such as self-renewal, capacity for differentiation, high proliferation, and capacity for forming tumors in xenografts.^6, 7^ Moreover, CD133^+^ cells were shown to be more resistant to radiation and standard chemotherapy than CD133^−^ cells.^45, 46^ Despite these studies, the regulation of CD133 expression and its role in tumorigenesis are still poorly understood. Here, we demonstrate both a novel mechanism of CD133 transcriptional regulation by p53 and CD133's role in tumor formation and proliferation. CD133 expression was inversely correlated with p53 expression in various cancer cell lines and tumor tissues from cancer patients. The inverse relationship between CD133 and p53 expression can be attributed to p53-mediated negative regulation of CD133 transcription; the CD133 promoter contains putative p53-binding sequences, and p53 suppresses the activity of the CD133 promoter. These sites were further shown to be critical for p53-mediated suppression of CD133. ChIP-qPCR analysis demonstrated that p53 directly binds the CD133 promoter under conditions of stress and recruits HDAC1 to the CD133 promoter, leading to epigenetic alterations in the p53-binding region. Cancer cell proliferation and capacity for transformation were impaired in the CD133 stable knockdown cell lines, which were accompanied by the suppression of stemness genes including NANOG, OCT4, SOX2, and c-MYC, suggesting that CD133 expression is required for tumor tissue growth and survival. Moreover, p53-mediated CD133 inhibition was required for the tumor-suppressive effects of p53 in several cancer cell lines. These results suggest that CD133 could be a potential target for tumor inhibition in highly tumorigenic cancers in which p53 function is impaired.

Various studies have shown that aberration of p53 expression can promote CSC initiation and progression.^26, 47^ Although these studies suggested that p53 could be a barrier to CSC formation, the precise mechanism used by p53 to regulate CSC survival and tumorigenesis was relatively unclear. The results presented here demonstrate that p53 can inhibit proliferation and tumor formation by suppressing CD133 transcription. Consistent with our observations, a recent study also showed that CD44 expression is directly regulated by p53. Moreover, p53-mediated inhibition of CD44 is required to suppress CSC initiation and capacity for tumor growth.^26^ Our data shows that p53 directly regulates CD133 transcription through binding to p53-binding motifs in the CD133 promoter, indicating that CD133 is a novel p53 target gene. In addition, CD133 levels are upregulated in highly tumorigenic cell lines such as NCCIT and NTERA2 in which p53 is diminished. These results provide evidence that p53 restrains the tumorigenic features of CSCs by modulating the expression of CSC-specific genes and suggest that CD133 could be a potential target for tumor inhibition in highly tumorigenic cancers in which p53 function is impaired.

In addition to our observation showing the regulation of CD133 expression by p53, CD133 has been demonstrated to be regulated by DNA methylation. The CpG island within the CD133 promoter P1-3 is hypermethylated in CD133^−^ glioma stem cells, and the treatment of human glioma cells with the demethylating agent 5-azacytidine leads to the rescue of CD133 expression.^48, 49^ Transforming growth factor beta (TGF-*β*) signaling has also been shown to induce CD133 expression and CD133^+^ cell population via demethylation of CpG sites on the CD133 promoters.^50, 51^ Consistent with this observation, blocking TGF signaling with arsenic trioxide (As2O3) attenuates the expression of CD133 in HCC.^52^ Our study identified the involvement of repressive histone modification in p53-mediated CD133 suppression. Growing evidence demonstrates that there is crosstalk between the DNA methylation and histone modification pathways. Several histone methyltransferases, including G9a, SUV39, and EZH2, can interact with DNA methyltransferases.^53, 54, 55^ Therefore, further investigation of crosstalk between these two epigenetic modifications required for the regulation of CD133 expression is needed.

Stem cell-like features are predictive factors for CSC tumorigenicity. Several studies have shown that CSCs highly express stemness genes such as NANOG, OCT4, SOX2, or c-MYC, which are critical for CSC tumor formation activity and survival.^35, 36, 37^ We demonstrated that depletion of CD133 suppresses the expression of stemness genes including NANOG, OCT4, SOX2, and c-MYC, and inhibits the cell growth and capacity for tumor formation of aggressive cancer cell lines. Although these results imply that CD133 may be associated with the expression of stemness genes in CSCs, the downstream mechanism of how CD133 regulates these stemness genes remains unclear. A recent study showed that CD133 can interact with p85 and activate the PI3K/Akt signal pathway, and this interaction results in the increased tumorigenic capacity of glioma stem cells.^56^ These results suggest that CD133 could have a role in modulating the expression of stemness genes in CSCs by interacting with several signal pathways; however, further investigation is required to understand the full potential of CD133 in CSC regulation.

Our study is the first to find that CD133 is a novel target gene of p53. These results reveal a previously unrecognized interaction between the p53 pathway and the CSC marker CD133, and suggest that the CD133–p53 interaction could be exploited as a potential target for CSC therapy. We anticipate that our results will improve the current understanding of how p53 regulates CSC initiation and progression.

## Materials and Methods

### Plasmids and constructs

The human CD133 promoter was cloned into the pG5 luciferase reporter vector, and the C/G to A/T mutation in the consensus p53-binding site was generated by site-direct mutagenesis. The human CD133 clone was obtained from the Korea Human Gene Bank (Medical Genomics Research Center, KRIBB, Daejeon, South Korea) and subcloned into the retroviral pBABE vector. All of the constructs were confirmed via sequencing. A set of lentiviral shRNAs against CD133 was purchased from Open Biosystems (shCD133-1(TRCN0000062143), shCD133-2(TRCN0000062144), and shCD133-3(TRCN0000062146)) (Open Biosystems, Huntsville, AL, USA). pCMV-Neo-Bam p53 R175H was a gift from Bert Vogelstein (Addgene plasmid # 16436, Cambridge, MA, USA).

### Cell culture

The cell lines used in this study were cultured in RPMI-1640 medium (NCCIT, LoVo, Jurkat, H1299, DLD1, HCT15, and SJSA1); McCoy's 5a medium (U2OS, HCT116, and SKOV3); Leibovitz's L-15 medium (SW480, MDA-MB-231, MDA-MB-468, and IMDM(K562)); F-12K medium (PC3, AGS, and A549); EMEM (HepG2, ACHN, SK-HEP-1, and IMR90); DMEM (A2058, 293T/17, Hela, MCF7, and NTERA2); or a 1 : 1 mixture of EMEM and F12 medium (SH-SY5Y), supplemented with 10% fetal bovine serum and 1% penicillin/streptomycin. HaCaT cells were cultured in DMEM supplemented with 10% BCS.

### Reagents and antibodies

For immunoblotting and immunofluorescence assays, we used anti-p53 clone DO-1 (Santa Cruz Biotechnology, CA, USA), anti-histone H3 (Santa Cruz Biotechnology), anti-PROM1 (CD133) (Abnova, Taipei, Taiwan), anti-Nanog, anti-Oct4, and anti-Sox2 (Abcam, Cambridge, UK); anti-c-Myc (Santa Cruz Biotechnology); anti-EZH2 (BD Biosciences, Franklin Lakes, NJ, USA); and anti-Actin (Millipore, Billerica, MA, USA) primary antibodies. For ChIP assay, we used agarose conjugated with anti-HA (Santa Cruz Biotechnology); anti-p53, anti-HDAC1, anti-H3K9me3, and anti-H3 acetylation (Millipore) antibodies. For protein immunoprecipitation assays, anti-p53 (Santa Cruz Biotechnology) and anti-HDAC1 (Millipore) were used. For the BrdU assay, anti-BrdU (Abcam) was used. Dox was purchased from Sigma-Aldrich (St Louis, MO, USA).

### Generation of stable cell lines

The retroviral p53 vector, CD133-expressing vector were cloned into the pBABE vector backbone. The lentiviral pLKO.1 vector encoding CD133 shRNA was purchased from Open Biosystems. The lentiviral pLKO.1 vector encoding CD133 shRNA was purchased from Open Biosystems and the retro- and lentiviruses were obtained from the 293T and 293FT cells, respectively, according to the manufacturer's protocol. PEG-it Virus Precipitation Solution (System Biosciences, CA, USA) was used to obtain concentrated virus. The NCCIT cells were infected with virus containing WT p53, WT CD133, or shRNA targeting CD133. Forty-eight  hours after infection, the cells were selected using puromycin (2 *μ*g/ml).

### Luciferase assays

NTERA2, NCCIT, and H1299 cell lines were seeded (2 × 10^4^) in six-well plates 24 h before transient transfection with 0.25 *μ*g of luciferase plasmid or 0.25–0.5 *μ*g of WT p53 plasmid using Lipofectamine2000 and LTX (Invitrogen, Carlsbad, CA, USA). The total amount of plasmid DNA was adjusted to 0.75 *μ*g with empty vector. Forty-eight hours following transfection, the luciferase activities of the cell lysates were determined according to the manufacturer's instructions (Promega, Madison, WI, USA). The relative luciferase activities were normalized to the whole protein contents of the cell extract.

### Immunoblot analysis

For immunoblotting, 20–40 *μ*g of protein extracts were boiled and separated by 11–13% SDS-polyacrylamide gel electrophoresis (SDS-PAGE). The proteins were transferred to polyvinylidene difluoride membranes, blocked for 1 h in Tris-buffered saline (TBS) containing 0.1% Tween 20 and 5% (w/v) dry skim milk powder, and incubated overnight with the indicated primary antibodies. The membranes were then incubated for 1 h with an HRP-conjugated secondary antibody (rabbit-HRP, Abcam; or mouse-HRP, Santa Cruz Biotechnology). Chemiluminescence was detected using chemiluminescence reagents (Animal Genetics, Tallahassee, FL, USA).

### Quantitative real-time PCR

Total RNA was isolated using Easy-blue reagent (Intron Biotechnology, Gyeonggi-do, Korea). Reverse transcriptase PCR was performed using the Access RT-PCR system (Promega). Quantitative real-time PCR was performed using the KAPA SYBR FAST qPCR kit (Kapa Biosystems, Wilmington, MA, USA) and a CFX96 real-time PCR detector (Bio-Rad, Hercules, CA, USA). GAPDH or 18 s rRNA was used to normalize gene expression levels for quantitative analyses.

### Nested PCR

Nested PCR primer sequences were used in nested amplification as previously described,^31^ using the PCR conditions indicated above.

### Immunocytochemistry

Immunocytochemistry was performed according to a standard protocol. Briefly, 2 × 10^4^ cells were seeded in a 12-well plate, fixed with 4% formaldehyde for 10 min at 37 °C, rinsed thoroughly with PBS, and blocked for 1 h with 1% BSA-PBS. Samples were incubated overnight at 4 °C with primary antibodies against CD133, p53, and stained with 4′,6-diamidino-2-phenylindole (DAPI). The secondary antibodies were then incubated for 1 h and samples were imaged using a Zeiss Axioplan I fluorescent microscope (Carl Zeiss, Oberkochen, Germany).

### Chromatin immunoprecipitation assay

ChIP assays were performed according to the manufacturer's protocol (Upstate Biotechnology, Lake Placid, NY, USA). A small portion of the cross-linked, sheared, chromatin solution was saved as the input DNA and the remainder was used for immunoprecipitation with an anti-HA-tag antibody conjugated to agarose (Santa Cruz Biotechnology). The immunoprecipitated DNA was purified with a PCR purification kit (QIAGEN, Venlo, The Netherlands) and analyzed by quantitative real-time PCR. Data are represented as the percentage of total input that was immunoprecipitated.

### Immunoprecipitation assay

Hela cells were collected and lysed in lysis buffer (40 mM HEPES (pH 7.4), 120 mM NaCl, 1 mM EDTA, 50 mM NaF, 1.5 mM Na_3_VO_4_, 10 mM *β*-glycerophosphate, 0.30% CHAPSO, and protease inhibitors) as previously described.^57^ The specific antibodies and beads were added to the supernatants and incubated overnight at 4 °C. The beads were then pelleted and washed twice with lysis buffer. The proteins were eluted in 2 × Laemmli buffer (Bio-Rad) or 4 × LDS sample buffer (Invitrogen) by boiling for 5 min. The immunoprecipitated proteins were used for immunoblotting.

### Cell growth assays

NCCIT (10^4^ cells) were grown on 60 mm plates with RPMI-1640 medium. Fresh medium was replenished every 3 days. Cells were counted at 1, 5, 9, and 12 days under a microscope using a hemocytometer.

### BrdU assay

BrdU assays were performed as previously described.^58^ NTERA2 or NCCIT cells were treated with BrdU (10 mM final concentration) for 1 h and then fixed with 4% PBS-based formaldehyde. The cells were then stained and counterstained with Alexa fluor 488 (Invitrogen).

### Soft agar colony forming assay

Cells (10^5^, 2 × 10^4^, and 10^4^) were seeded in six-well plates containing 0.4% Noble top agar and incubated at 37 °C for 14–21 days. Fresh medium was supplemented every 3 days. Colonies were visualized by Nitro Blue Tetrazolium staining and counted. Values represent at least three independent experiments.

### Adherent colony formation assay

Cells (5 × 10^4^) were seeded in six-well plates with culture medium and incubated at 37 °C for 14 days, stained with 0.05% crystal violet solution and counted. Values represent at least three independent experiments.

### Clinical samples

A total of five hepatocellular carcinoma tissues and corresponding normal tissues were obtained from Yonsei University, School of Medicine, Seoul, Korea. The patients included five men aged 43–58 years. Liver tissue resections for HCC occurred between 1999 and 2000. Patient clinical information and tumor grades are summarized in Supplementary Table 2. This study was approved by the Institutional Review of Board of the Songeui Campus, College of Medicine, The Catholic University of Korea (IRB approval number; CUMC10U036), and written informed consent was obtained from all subjects.

### Statistical analysis

Statistical significance was analyzed using Student's *t*-test and expressed as a *P*-value. All data represent at least three independent experiments.

## Abbreviations

CSC: cancer stem cell
HDAC: histone deacetylase
OCT4: octamer-binding transcription factor 4
DNA: deoxyribonucleic acid
TSA: trichostatin A
SAHA: suberoylanilide hydroxamic acid
Dox: Doxorubicin
5-FU: 5-fluorouracil
MCF-7: Michigan Cancer Foundation-7
IF: immunofluorescence
HCC: hepatocellular carcinoma
UV: ultraviolet
mRNA: messenger RNA
qRT-PCR: quantitative reverse transcription polymerase chain reaction
siRNA: short interfering RNA
P1: promoter 1
PCR: polymerase chain reaction
WT: wild-type
ChIP: chromatin immunoprecipitation
BrdU: bromodeoxyuridine
shRNA: short hairpin RNA
EZH2: Enhancer of zeste homolog 2
DAPI: 4′,6-diamidino-2-phenylindole
