# Supplementary material for: Transcriptional repression of cancer stem cell marker CD133 by tumor suppressor p53
Source: Cell Death Dis. 2015 Nov 5;6(11):e1964–. doi: 10.1038/cddis.2015.313 (PMC4670923; doi:10.1038/cddis.2015.313)
Supplement: Supplementary Information [file cddis2015313x1.docx]

**Supplementary Figures**

Figure S1 Expression pattern of CD133 and p53 in various cancer cell lines. Expression levels of CD133 and p53 were determined using immunoblot analysis in 27 cancer cell lines and quantified by Image-J software.

Figure S2 Overexpression of mutant type p53. Western blot was performed using lysates of LoVo cells transfected with mutant p53(R175H) expressing vector for the indicated time.

Figure S3 The structure of CD133 promoter region containing the wild type or mutated p53 consensus DNA binding sites for luciferase assay. (a) 5’ UTR promoter region of human CD133 and each exon (Exon A, B, C1-3 cluster of 1C, D1-4 cluster of 1D and E1-4 cluster of 1E) are alternatively spliced to Exon2. P1, P2, P3, P4 and P5 present alternative promoter for CD133 gene. (b) The consensus sequence of p53 and potential p53 binding site in the CD133 promoter 1 region, 971-750 bp (WT1) or 714-693bp (WT2) upstream from the transcription start site, and site direct mutated p53 binding site (MT1 and MT2). (c) Normal CD133 promoter or mutated CD133 promoter activity in NCCIT cell lines. The cells were treated with 0.5 µg/ml of doxorubicin after cotransfected with reporter gene vector. The error bars represent the mean ± s.d. *n*=3.

Figure S4 Physical Interaction of p53 and HDAC1. (a) ChIP analysis of HDAC1 binding to CD133 promoter DNA or enrichment of H3Ac and H3K9me3 in H1299 cells transfected with either empty or p53 expressing vector. The percentage of the total promoter isolated by ChIP was normalized with 5% input DNA and determined by quantitative PCR. (b) Immunoblot analysis of p53 immunoprecipitates (left panel) and HDAC1 immunoprecipitates (right panel) in the lysate of Hela cells. Antibodies used for IP are indicated. The error bars represent the mean ± s.d. *n*=3. *p<0.05, **p<0.01, and ***p<0.001.

Figure S5 CD133 knockdown greatly down regulates stemness gene and tumor cell proliferation. (a) BrdU assay was performed for determine CD133 proliferation ability in the control NCCIT cell or CD133 knock down NCCIT cell lines. Scale bar, 50μm. (b) Soft agar colony formation assay in control or CD133 knock down stable NCCIT cells (10^5^ cells). Two weeks after seeding, colonies were counted. Graph show quantification of colonies. Scale bar, 250 μm The error bars represent the mean ± s.d. *n*=3.
