# Supplementary material for: Transcriptional repression of cancer stem cell marker CD133 by tumor suppressor p53
Source: Cell Death Dis. 2015 Nov 5;6(11):e1964–. doi: 10.1038/cddis.2015.313 (PMC4670923; doi:10.1038/cddis.2015.313)
Supplement: Supplementary Tables [file cddis2015313x2.docx]

**Table 1. Genomic p53 status of 28 human cancer cell line.**

| **cell** | **Tumor** | **Type** | **p53** | **Zygosity** | **Mutation** |
| --- | --- | --- | --- | --- | --- |
| SH-SY5Y | T | neuroblastoma | WT |  |  |
| A2058 | T | melanoma | MT | Homozygous | p.V274F |
| 293T/17 | T | epithelial SV40 T antigen | WT |  |  |
| HeLa | T | adenocarcinoma | WT |  |  |
| HaCaT | N | epithelial keratinocyte | MT | Heterozygous | p.H179Y/p.R282W |
| Hep G2 | T | hepatocellular carcinoma | WT |  |  |
| ACHN | T | renal cell adnocarcinoma | WT |  |  |
| SK-HEP-1 | T | adenocarcinoma | WT |  |  |
| IMR90 | N | fibroblast | WT |  |  |
| MDA-MB-231 | T | adenocarcinoma | MT | Homozygous | p.R280K |
| MDA-MB-468 | T | adenocarcinoma | MT | Homozygous | p.R273H |
| SW-480 | T | colorectal adenocarcinoma | MT | Heterozygous | p.R273H/p.P309S |
| Jurkat | T | acute T cell leukemia | MT | Heterozygous | p.T256A/p.D259G/  p.S260A |
| MCF7 | T | adenocarcinoma | WT |  |  |
| K-562 | T | chronic myelogenous leukemia (CML) | MT | Homozygous | p.Q136fs*13 |
| SK-OV-3 | T | adenocarcinoma | MT | Homozygous | p.S90fs*33 |
| U2OS | T | osteosarcoma | WT |  |  |
| H1299 | T | carcinoma; non-small cell lung cancer | MT | Homozygous | p53 null |
| PC3 | T | adenocarcinoma | MT | Homozygous | p53 null |
| A549 | T | carcinoma | WT |  |  |
| AGS | T | gastric adenocarcinoma | WT |  |  |
| LoVo | T | colorectal adnocarcinoma | WT |  |  |
| NCCIT | T | pluripotent embryonal carcinoma, teratocarcinoma | MT | Homozygous | p.V272fs*73 |
| NTERA2 | T | malignant pluripotent embryonal carcinoma | WT |  |  |
| HCT116 | T | colorectal carcinoma | WT |  |  |
| DLD-1 | T | colorectal adenocarcinoma | MT | Homozygous | p.S241F |
| HCT-15 | T | colorectal adenocarcinoma | MT | Heterozygote | WT/p.P153A |
| SJSA-1 | T | osteosarcoma; multipotential sarcoma | WT |  |  |

The p53 mutation patterns of 28 human cancer cell lines used in this study (Figure 1c and Supplementary Figure 1). MT = mutated; WT = wild type.

**Table 2. Information of human tissue samples (HCC).**

| **Tissue No.** | **Grade** | **Background** | **Age** | **Gender** |
| --- | --- | --- | --- | --- |
| 35 | 2>3 | Cirrhosis | 56 | M |
| 57 | 2>3 | Cirrhosis | 44 | M |
| 68 | 3 | Cirrhosis | 43 | M |
| 77 | 3 | Cirrhosis | 58 | M |
| 80 | 3 | Cirrhosis | 43 | M |

Liver tumor and normal tissue were obtained from five patients. Tissue numbers of each sample, tumor grades, background of samples, patient ages and genders are listed.
