# Supplementary figures and images for: Transcriptional repression of cancer stem cell marker CD133 by tumor suppressor p53
Source: Cell Death Dis. 2015 Nov 5;6(11):e1964–. doi: 10.1038/cddis.2015.313 (PMC4670923; doi:10.1038/cddis.2015.313)

Supplementary Figure 1

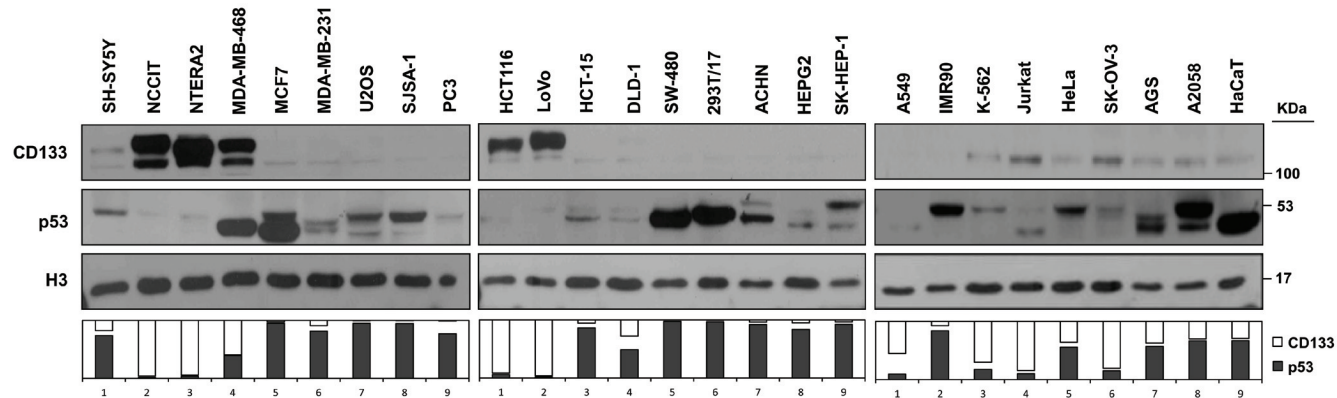

Supplement: Supplementary Figure 1 [file cddis2015313x3.pdf]

Supplementary Figure 2

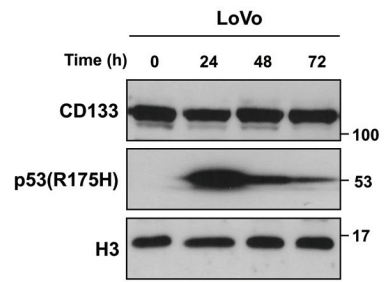

Supplement: Supplementary Figure 2 [file cddis2015313x4.pdf]

a

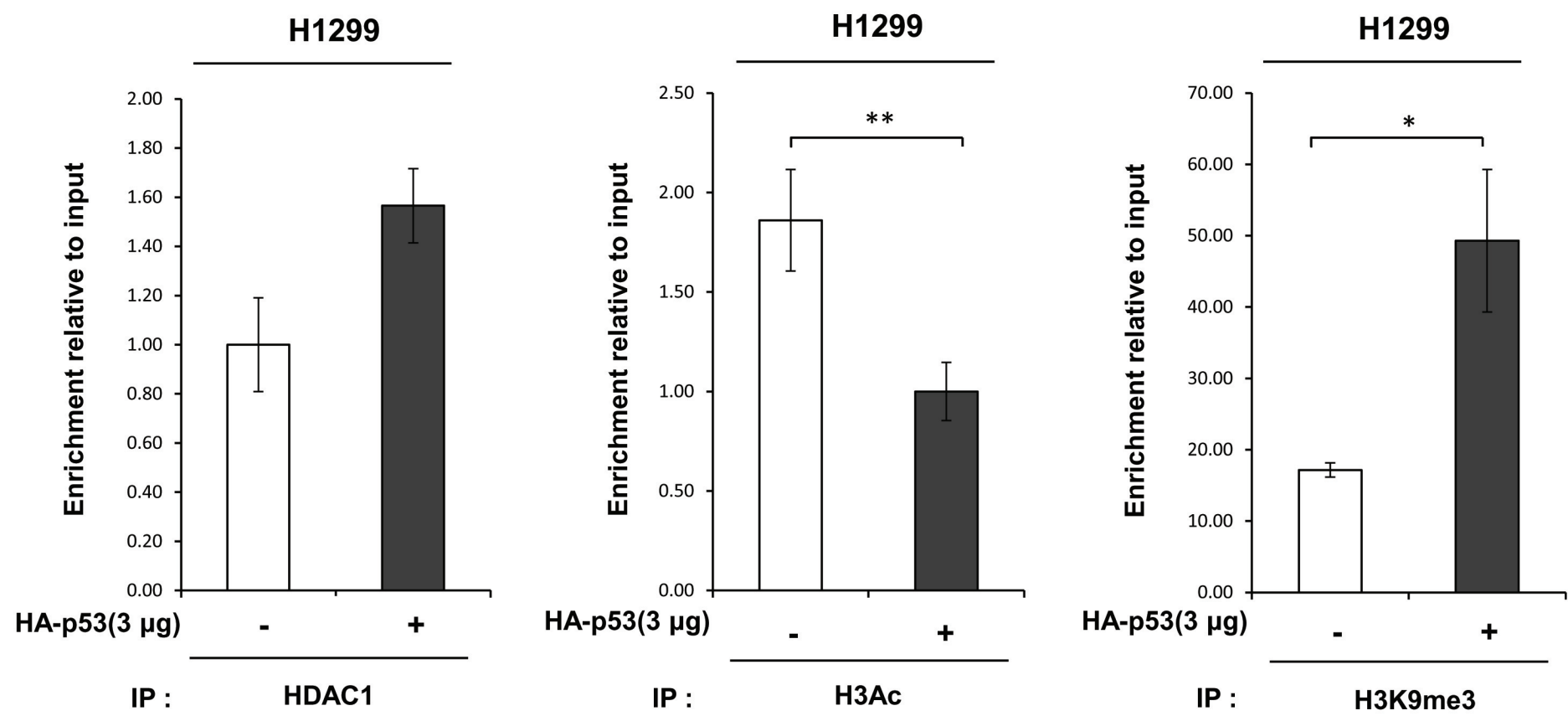

b

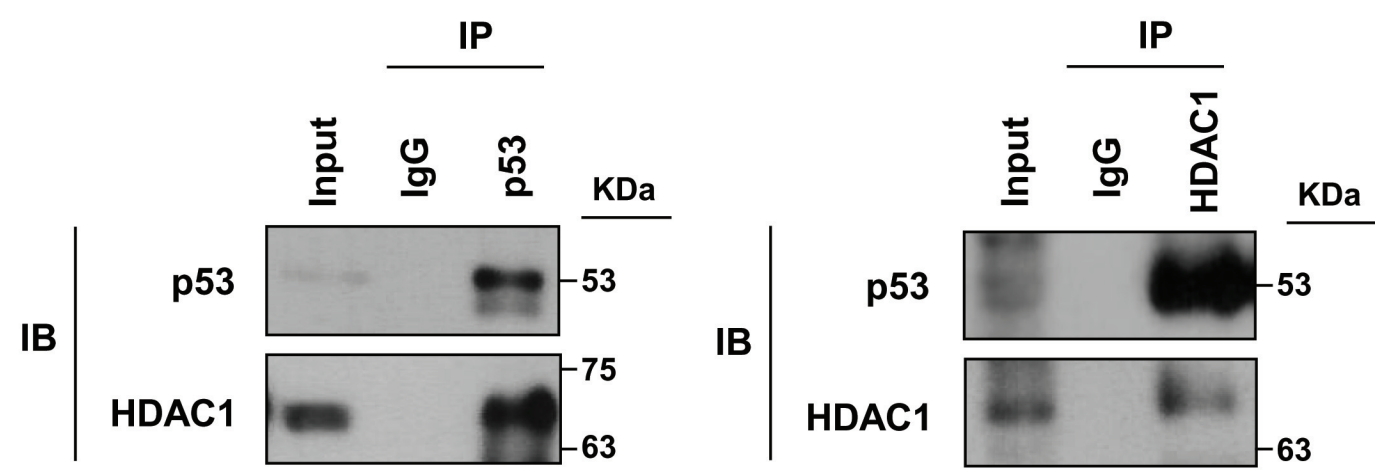

Supplement: Supplementary Figure 4 [file cddis2015313x6.pdf]

**Supplementary Figure 5**

**a**

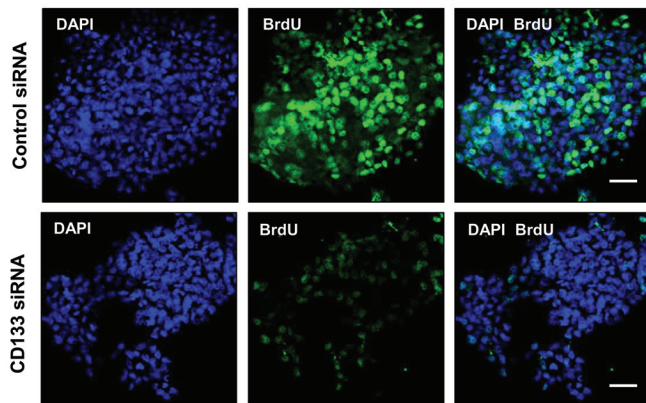

**b**

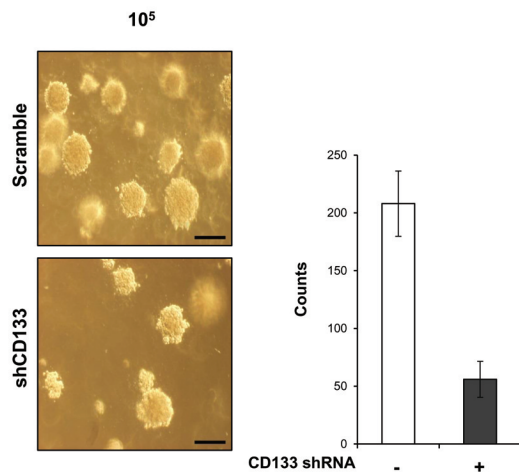

**c**

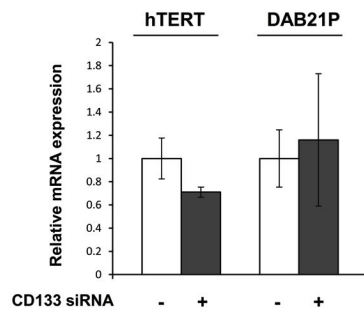

Supplement: Supplementary Figure 5 [file cddis2015313x7.pdf]
