# Supplementary material for: Transcriptional repression of cancer stem cell marker CD133 by tumor suppressor p53
Source: Cell Death Dis. 2015 Nov 5;6(11):e1964–. doi: 10.1038/cddis.2015.313 (PMC4670923; doi:10.1038/cddis.2015.313)

Supplementary Figure 3

a

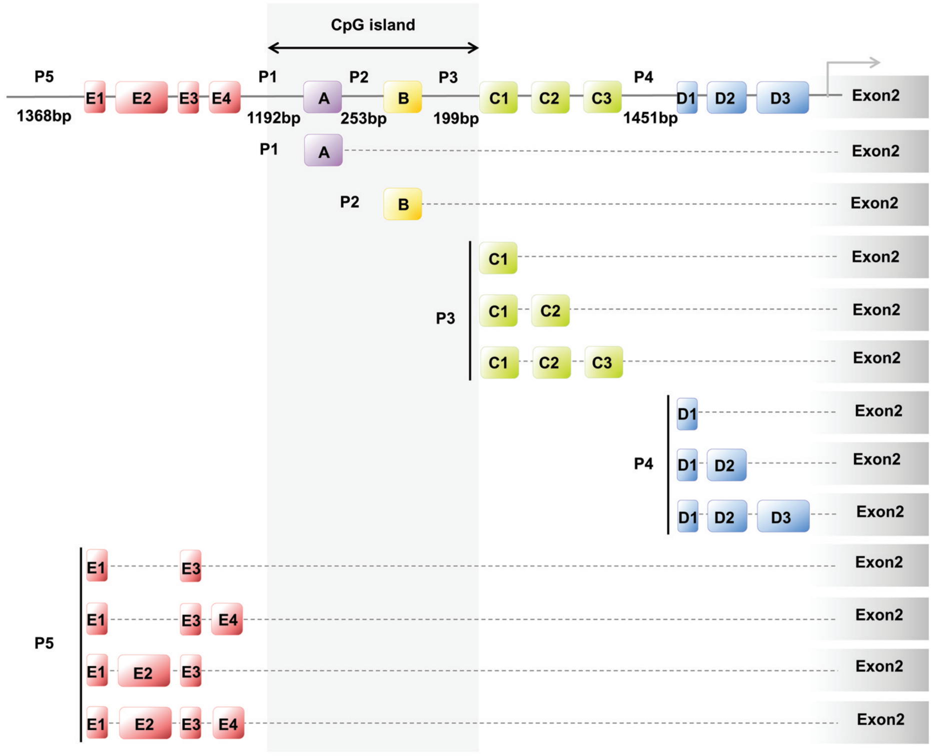

b

Canonical p53 binding site

PuPuPuC(A/T) (A/T)GPyPyPy

Non-canonical p53 binding site in CD44 promoter

CCAGT G GGGCT CGG AGGCA C AGGCA

Potential p53 binding site in CD133 promoter

*WT1
(-971) AGCCAAACCA TCT AACCT TGCCA (-750)
*MT1
(-971) ATCCA AACTA TCT ATCCT TGCAA (-750)

*WT2
(-714) AAACA C CTGCT GT ATGCT AGACA (-690)
*MT2
(-714) AAATA C CTGAT GT ATGAT AGATA (-690)

c

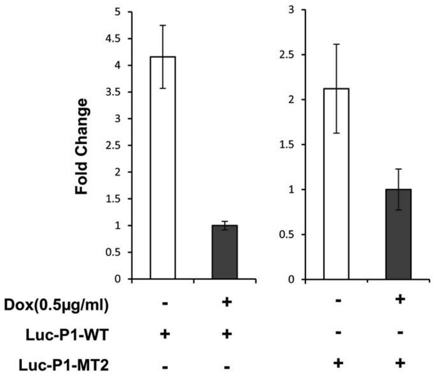

Supplement: Supplementary Figure 3 [file cddis2015313x5.pdf]
